# Multiple Virus Infections in Western Honeybee (*Apis mellifera* L.) Ejaculate Used for Instrumental Insemination

**DOI:** 10.3390/v11040306

**Published:** 2019-03-29

**Authors:** Jana Prodělalová, Romana Moutelíková, Dalibor Titěra

**Affiliations:** 1 Department of Virology, Veterinary Research Institute, Hudcova 296/70, 621 00 Brno, Czech Republic; moutelikova@vri.cz; 2 Department of Zoology and Fisheries, Faculty of Agrobiology, Food and Natural Resources, Czech University of Life Sciences Prague, Kamýcká 129, 165 00 Prague, Czech Republic; titera@beedol.cz

**Keywords:** honeybees, ejaculate, instrumental insemination, virus detection, BQCV, SBV

## Abstract

Instrumental insemination of *Apis mellifera* L. queens is a widely employed technique used in honeybee breeding that enables the effective control of mating. However, drone semen represents a potential source of honeybee viruses. In this study, 43 semen doses collected from apparently healthy drones, and consequently used in instrumental insemination, were analysed using PCR or RT-PCR to detect the presence of viral genome of 11 honeybee viruses. In 91% of samples, viral infection was detected. The survey revealed genomes of five viruses, namely Deformed wing virus (DWV), Acute bee paralysis virus (ABPV), Black queen cell virus (BQCV), Sacbrood virus (SBV), and *A. mellifera* filamentous virus (*Am*FV) in 84%, 19%, 14%, 2%, and 67% of samples, respectively. Single infection (30% of samples) as well as multiple infection (61% of samples) of two, three or four pathogens were also evaluated. To the best of our knowledge, this is the first study describing the presence of the BQCV and SBV genome sequence in drone ejaculate. Phylogenetic analysis of BQCV partial helicase gene sequence revealed the high similarity of nucleotide sequence of described Czech strains, which varied from 91.4% to 99.6%. The findings of our study indicate the possibility of venereal transmission of BQCV and SBV.

## 1. Introduction

Instrumental insemination of *Apis mellifera* queens started in the 1920s [1], and was first described in the 1940s [2]. It has been widely used in honeybee breeding programs and represents a primary method of controlling mating where there is no other option to effectively isolate a breeding population [3]. As honeybee queens mate in flights with drones originating from colonies up to 15 km distant [4], the use of geographically isolated mating stations usually located in small islands or confined valleys is the second and the last method for honeybee breeding [4,5]. Naturally, honeybee queens mate with numerous drones (12–14 on average) coming from various genetic sources, which is generally considered to be a means to increase colony fitness [6,7,8,9]. However, techniques enabling the mixing of large sperm volumes to inseminate the queens have been described [10,11]. Another significant advantage of instrumental insemination is the ability to not only store but also ship semen instead of live bees, which minimizes the risk of spreading pests and diseases threatening the health and vigour of honeybee colonies [1]. Nevertheless, honeybee viruses in semen have been recognized among the risk factors in the trade of honeybees and their products, as collected semen and consequently semen trade could be responsible for virus spreading [12]. Actually, honeybee viruses have been identified as crucial contributors to huge losses of managed honeybees, which have been reported in the recent past worldwide [13]. Moreover, queen loss has been mentioned as the second most important factor in colony collapses in Europe [14]. Queen health is a crucial factor affecting the survival of a colony. Despite queens being considered less susceptible to infection than workers, several viruses cause problems for queen bees [15]. Regardless of the fact that the knowledge of these viruses grows every year, little is known about the transmission of viruses to the honeybee queen during instrumental insemination. However, the presence of several viruses in ejaculate, a prerequisite of virus transmission, has been described [16,17,18,19]. Beekeeping represents one of the basic fields of agriculture. In the Czech Republic, 56,921 beekeepers keeping 662,523 colonies were registered in 2016. Moreover, in the Czech territory there were 67 queen bee producers rearing 33,443 queen bees annually [20]. Instrumental insemination is employed in approximately 2‒5% of all produced queens in the Czech queen-rearing farms [21]. However, viral contamination of the used ejaculate has not been tested yet. Therefore, the presence of viral nucleic acids of 11 honeybee viruses was surveyed in this study.

## 2. Materials and Methods

### 2.1. Sample Collection and Handling

In 2016 and 2017, a total of 43 pooled semen doses of Austrian (2), Czech (33), Hungarian (3), and German (5) origin were collected. Each dose contained ejaculate obtained from 10 drones per colony. Active drones aged two weeks were collected from flight cages placed in apparently healthy colonies showing no signs of overt viral infection. Moreover, the sampled colonies were regularly tested for the presence of *Paenibacillus larvae*, the causative agent of American foulbrood (all negative); the presence of ectoparazitic mite *Varroa destructor* was also monitored. Colony conditions were evaluated by the beekeeper based on direct observation. All semen doses included in the survey were prepared using a protocol for instrumental insemination [21]. Briefly, ejaculate was taken directly from the drone’s penis. Ejaculation was induced by pressing on the thorax and semen was collected in a disposable glass capillary connected with a vacuum device. Semen doses were stored at room temperature until use. The period of storage of the ejaculate at room temperature typically ranges from 5 to 36 h. In rare cases (e.g., parental combinations from multiple sites), ejaculate can be kept for up to five days, but longer storage results in a decrease in sperm counts in the spermatheca of inseminated queens. Prior to instrumental insemination, 3 to 5 μL of each semen dose were sampled and stored at −80 °C until nucleic acid extraction.

### 2.2. Nucleic Acid Preparation

Both DNA and RNA were extracted simultaneously using magnetic beads. Chemagic Viral DNA/RNA Kit (Perkin Elmer Chemagen, Baesweiler Germany) was employed according to the slightly modified manufacturer’s protocol. In short, immediately before nucleic acid extraction, the semen samples were homogenised directly in a lysis buffer supplied with the extraction kit in the presence of Garnet Beads 0.70 mm (Qiagen, Hilden, Germany) by vortexing (3500 rpm/2 min). Homogenates were centrifugated for 1 min at 13,000 rpm and 200 μL of the supernatants were mixed with 30 μL of magnetic beads. Subsequently, the manufacturer’s protocol was continued. The obtained nucleic acids were stored at −80 °C until further use.

### 2.3. Molecular Detection of Viruses and Sequence Analysis

For the detection of RNA viruses, a random primed reverse transcription (RT) was carried out with the use of ProtoScript® II First Strand cDNA Synthesis Kit (New England Biolabs, Ipswich, MA, USA) according to the manufacturer’s protocol. Target viral sequences were detected by PCR with specific primers employing Aptamer Hot Start Master Mix (Top-Bio, Prague, Czech Republic. The primers were chosen on the basis of previous virus survey in the Czech apiaries. The used primers [16,22,23,24,25,26,27,28,29,30,31,32,33], sizes of the expected amplicons, and references are listed in Table S1. The presence of viral RNA or DNA of the following honeybee viruses was surveyed: Deformed wing virus A and B (DWV), Acute bee paralysis virus (ABPV), Israeli acute paralysis virus (IAPV), Kashmir bee virus (KBV), Black queen cell virus (BQCV), Sacbrood virus (SBV), *Varroa destructor* macula-like virus (*Vd*MLV), Big Sioux River virus (BSRV), Lake Sinai virus (LSV), Chronic bee paralysis virus (CBPV), and *Apis mellifera* filamentous virus (*Am*FV). The specificity of detection primers was verified on our own field isolates of honeybee viruses. The PCR products were examined by electrophoreses in a 1.5% agarose gel stained with Midori Green stain (Nippon Genetics Europe, Dueren, Germany) and visualized by ultraviolet transillumination. Selected PCR products of employed diagnostic assays were submitted to sequencing (Eurofins, Ebersberg, Germany) and the obtained sequences were analysed with the use of MEGA version 7 [34]. The dendrograms were prepared with the neighbour-joining method and the evolutionary distances were calculated with the use of the Kimura 2-parameter model [35]. The BQCV sequences described in this study were submitted to the GenBank under accession numbers MG584538, MH992095, MH992096, and MH992097 (strains Dol826, Dol35, Dol45, and Dol47). Likewise, phylogenetic analyses of the only SBV strain (GenBank accession number MK550481) detected in a drone ejaculate was conducted and a dendrogram was prepared with the use of the same tools as in BQCV analyses. Accession numbers of the described Czech strains as well as other analysed strains of BQCV and SBV are listed in Table S2.

## 3. Results

**Figure 1:**
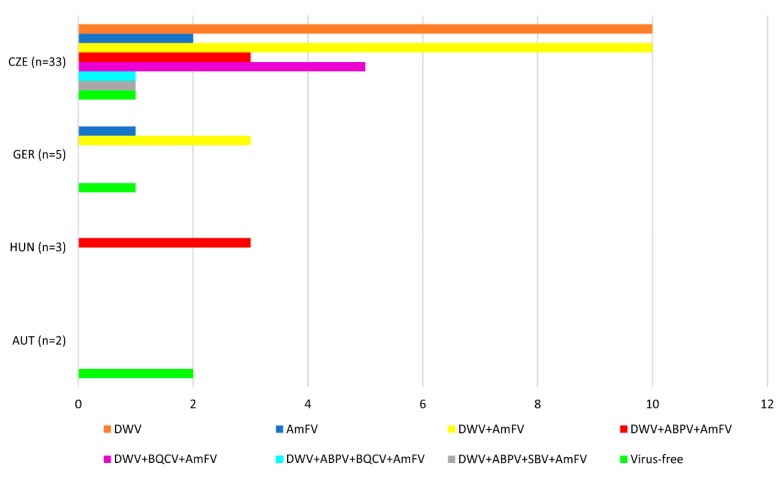
Schematic illustration of numbers of drone ejaculate samples positive for one or more honeybee viruses. Samples are grouped according to the country of the sperm collection.

In total, 43 pooled drone semen samples were surveyed for the presence of 11 honeybee viruses. The screening revealed genomes of five viruses, namely DWV, ABPV, BQCV, SBV, and *Am*FV. Within the detected viral species, DWV and *Am*FV were discovered as the most prevalent viruses in the analysed samples. Viral infection was detected in 91% (*n* = 39/43) of samples. Single infection (30% of samples; *n* = 13/43) as well as multiple infection (61% of samples; *n* = 26/43) of two, three or four pathogens were also evaluated. The results including pathogen combinations are summarized in Figure 1. Only 9% (*n* = 4/43) of samples were free of all tested viruses. Detailed description of detected viral pathogens in individual samples and summary of multiple infections are described in Tables S3 and S4.

**Figure 2:**
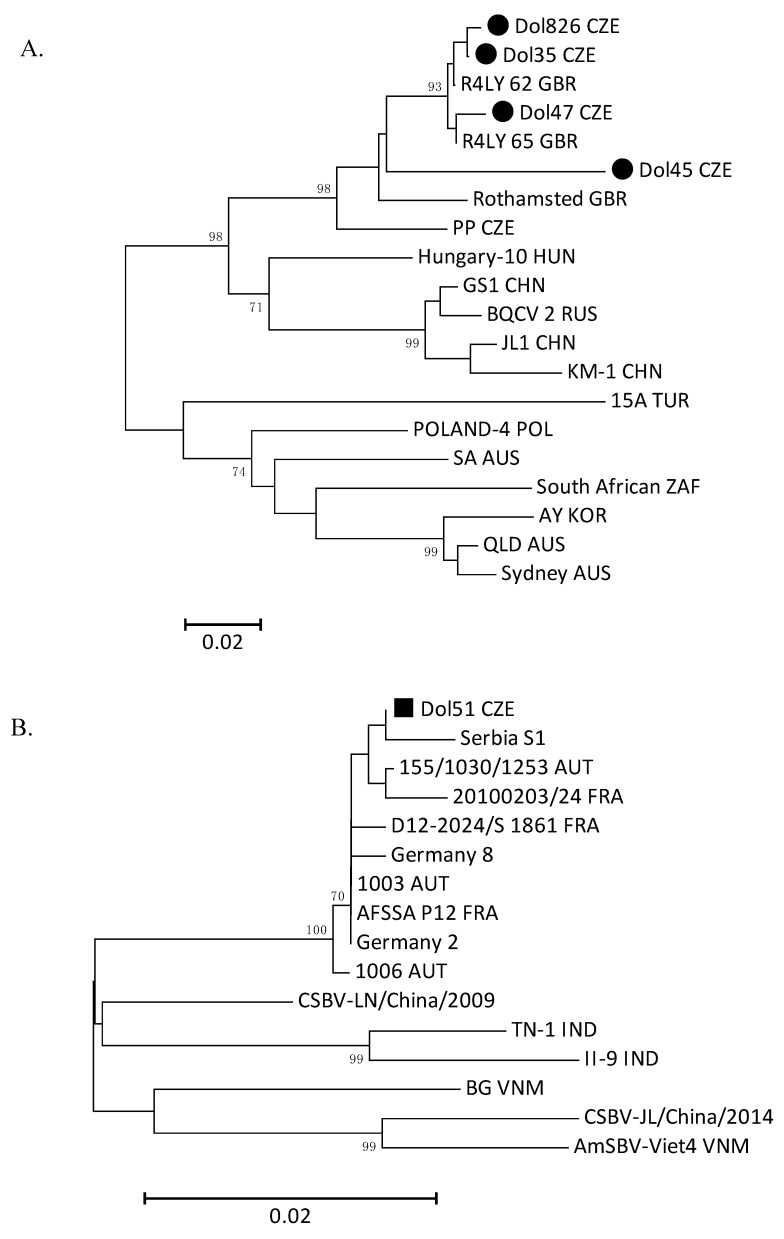
(**A**) Phylogenetic tree based on BQCV partial helicase gene sequences (between nt positions 2679 and 2961 referring to the complete genome sequence KY243932). The Czech BQCV strains described in this study are marked with a black dot (●). (**B**) Phylogenetic tree based on partial SBV polyprotein gene sequences (between nt 240 and 668 referring to the complete genome sequence HM237361). The Czech SBV strain from this study is marked with a black square (■). The evolutionary distances in both trees are in the units of the number of base substitutions per site. The trees were generated with the neighbour-joining method using MEGA version 7. Bootstrap values (1000 replicates) below 70% were hidden.

The obtained amplified sequences of BQCV partial helicase gene (between nt positions 2679 and 2961 referring to the complete genome sequence KY243932) were compared to the available BQCV sequences (see Table S2). Phylogenetic analysis revealed high similarity of nucleotide sequence of the described Czech strains, which varied from 91.4% to 99.6%. Constructed phylogenetic tree (Figure 2A) showed that the Czech strains Dol826, Dol35, and Dol47 were closely clustered with the British BQCV strains R4LY62 and R4LY65 (98.6–99.6% of nt similarity). The last Czech strain Dol45 created a separate branch (92.3–92.7% similarity with both British strains). Another available Czech sequence of BQCV (strain PP) showed 90.1–93.6% similarity with our described strains. The most distant BQCV strain 15A from Turkey demonstrated nt similarity rate of 73.4–79.0%. Likewise, to verify SBV detection, the obtained amplicon was sequenced and the occurrence of SBV genome in the drone ejaculate sample 51/17 (2.3%, *n* = 1/43) was confirmed). This sequence of SBV partial polyprotein gene (nt position 240–668 referring to the complete genome sequence HM237361) was aligned with all partial European sequences of SBV and also with several Asian complete sequences of SBV available in the GenBank (see Table S2). The phylogenetic analysis (Figure 2B) showed very high similarity between all European SBV strains in the analysed partial sequence (99.1–99.8% similarity on nucleotide level).

## 4. Discussion

Only limited data exist on the effect of stressors on health and quality of reproductive casts of honeybees and the available studies are historically focused on queens. Currently, drone health and quality are presented as vital aspects of reproduction management [36]. However, the virological status of drones is not usually monitored. Honeybee viruses are transmitted vertically from a mother to her offspring, or horizontally among individuals of the same generation. In eusocial insects, horizontal transmission can occur in multiple ways, either directly through contact between individuals, contaminated colony food, and infected drone sperm (i.e., venereal transmission), or indirectly through biological vectors such as the parasitic mite *Varroa destructor* [37]. In the present study, we demonstrate the occurrence of viral genome of DWV, ABPV, BQCV, SBV, and *Am*FV in 91% (*n* = 39/43) of the ejaculate doses tested. Similarly, sequences of viral nucleic acid of DWV, ABPV, IAPV, and *Am*FV were detected in the semen of apparently healthy drones [16,17,18,19], which implies the possibility of venereal transmission. Furthermore, sexual transmission of DWV by infected drones was clearly demonstrated [26,38], and the virus can be consequently transovarially transmitted through infected queen gonads to eggs [38]. Both natural mating and artificial insemination were found to be effective transmission routes of DWV [38,39]. Furthermore, the importance of venereal transmission during natural mating or instrumental insemination is supported by the detection of several honeybee viruses in fertilised queen eggs [40] when the presence of DWV, ABPV, and BQCV in 40%, 14%, and 5% of eggs, respectively, was described. On the contrary, BQCV (+ssRNA virus within family *Dicistroviridae*), one of the honeybee viruses most often detected with laboratory techniques in adult honeybees worldwide [41], has not been described in ejaculate yet; and venereal transmission was not ascertained. However, the vertical transmission of the virus from an infected queen to her progeny was demonstrated [40]. The presence of BQCV was confirmed in colony food, such as pollen and honey, as well as in the gut, which provides an evidence for the horizontal transmission of the virus via food-borne infection [37]. Although BQCV is most often isolated from asymptomatic colonies, overt infection causes deaths of queen pupae and pre-pupae, which are being found decomposed in patchily black cells [42]. BQCV infection was suggested to be associated with colony weakening [43]. However, it is deleterious, especially on queen-rearing farms [44]. Thus, the detection of BQCV genome sequences in 13.9% (*n* = 5/43) of semen doses obtained from apparently healthy drones and subsequent use of the sperm for instrumental insemination of a bee queen could imply future risk to the colony health via sold queen. However, in order to assess the effect of insemination with the contaminated ejaculate, the life story of these colonies will have to be monitored. Interestingly, one ejaculate sample was found to be positive for SBV, which is a causative agent of sacbrood disease. The virus is transmitted horizontally to young larvae through food contaminated by glandular secretion of latently infected nurse bees. The highest prevalence of sacbrood disease is observed during early spring, probably due to cold stress induced by fluctuating temperatures [45]. Although SBV is frequently detected in managed European honeybee colonies [46,47,48,49,50,51], only one of 43 ejaculate samples was tested positive for this virus. Even though infection is lethal for drone and worker brood, infected colonies usually survive, and thus SBV poses only a moderate threat to managed European honeybees [13]. On the other hand, SBV has been recognized as a dangerous pathogen of the Asian honeybee (*Apis cerana*) capable of causing huge losses in the Asian apiculture industry [45]. SBV was also found to be infecting adult queens; however, the effect of the infection on behaviour and physiology of queens was not clarified [52]. Since SBV may primarily be evolutionary adapted to the horizontal transmission route [53], the impact of venereal transmission of SBV is probably negligible. Among 43 tested samples, only four samples were free of all surveyed viruses. Based on the available data, we were not able to identify any differences between infected and those virus-free colonies (two Austrian, one German, and one Czech origin). These results are, however, in accordance with previously published data describing only a minor fraction of the honeybee populations free of persistent virus throughout the year [46,47,48].

In conclusion, we demonstrated the presence of BQCV and SBV partial genome sequences in semen doses used in instrumental insemination of honeybee queens. Thus, our results indicate the possibility of venereal transmission of those two important honeybee pathogens. The use of ejaculate obtained from infected drones could represent an effective model of BQCV’s transmission and the virological analysis of the collected semen testing could help to maintain the high quality of produced queen bees.

## Supplementary Materials

The following are available online at https://www.mdpi.com/1999-4915/11/4/306/s1, Table S1: List of PCR primers used in the study. Table S2: Accession numbers of BQCV strains used in phylogenetic analysis of partial helicase gene segment. Table S3: List and description of samples and detection of viral sequences in sperm used for insemination. Table S4: The distribution of honeybee viruses in single or mixed infections in drone semen samples.
