# Supplementary material for: Multiple Virus Infections in Western Honeybee (Apis mellifera L.) Ejaculate Used for Instrumental Insemination
Source: Viruses. 2019 Mar 29;11(4):306. doi: 10.3390/v11040306 (PMC6521257; doi:10.3390/v11040306)
Supplement: Supplementary file 1 [file viruses-11-00306-s001.zip › viruses-467571 supplementary proof/Table S1.pdf]

Table S1: List of PCR primers used in the study.

Primer designation	5‘ – 3‘ sequence	Reference	Amplicon size (bp)
DWV-1 20f<br>DWV-1 157r	CGAATTACGGTGCAACTAAC<br>ACAATAGATGGTCGGTGACA	[a]	559 bp
DWV-4 2345f<br>DWV-4 2779r	ATTGTGCCAGATTGGACTAC<br>AGATGCAATGGAGGATACAG	[a]	435 bp
DWV-10 8934f<br>DWV-10 9599r	CCTATCGAGCTGCACGACTT<br>CCGAGACCTTGTCCAGGTTA	[a]	666 bp
Pan-DWV-F<br>Pan-DWV-R	ACGCAACCCCAGGAAT<br>GTAGCTAATTTTACCCAATCTTTAAA	[b]	179 bp
ABPV-1 5381 f<br>ABPV-1 5517 r	AATGGGCCTATGGACTTTTCTA<br>AAATCTCCTGCAATAACCTTGG	[c]	178 bp
ABPV 1F<br>ABPV 2R	AGCCACTATGTGCTATCGTAT<br>ATGGTGACCTCTGTGTCATTA	[d]	207 bp
BQCV 2144+<br>BQCV 2726-	TGAGAGCTGCAGAACAAGAG<br>TCACGTGTCAAGGCTTCATC	[e]	583 bp
BQCV 3F<br>BQCV 4R	GCAAGCTCTTCCAATGATAG<br>AAGATTCAGCCGAGTCCTTA	[d]	322 bp
SB 1F<br>SB 2R	ACCAACCGATTCCTCAGTAG<br>CCTTGGAACTCTGCTGTGTA	[f]	487 bp
*Am*FV rrSSU F<br>*Am*FV rrSSU R	ACGAACGACTATCTAGCCATGAAC<br>GTCCGTTTCGGAGTGCATGAC	[g]	591 bp
*Am*FV TS F<br>*Am*FV TS R	CGCATGTACCAACAACTCGTAC<br>CACAGTTGGTGTAGCGCAGT	[g]	361 bp
BSRV-4714 f<br>BSRV-37 r	RGTGCAGCTTTATGCGTTGCC<br>CCGCTGTTGAGAATAAGGATATCCAGG	[h]	519 bp
LSVdeg f<br>LSVdeg r	GCCWCGRYTGTTGGTYCCCCC<br>GAGGTGGCGGCGCSAGATAAAGT	[i]	578 bp
VdMLV f<br>VdMLV r	ATCCCTTTTCAGTTCGCT<br>AGAAGAGACTTCAAGGAC	[j]	438 bp
IAPV IGR f<br>IAPV IGR r	CGATGAACAACGGAAGGTTT<br>ATCGGCTAAGGGGTTTGTTT	[k]	766 bp
KBV2 6639 f<br>KBV2 6801 r	CCATACCTGCTGATAACC<br>CTGAATAATACTGTGCGTATC	[c]	200 bp
CBPV 1-1<br>CBPV 1-2	TCAGACACCGAATCTGATTATTG<br>ACTACTAGAAACTCGTCGCTTCG	[l]	570 bp
CBPV A7<br>CBPV A8	ACTCCCGTCGTTGTGTTCTC<br>GGCGATTGGTATTTGTTTGG	[m]	895 bp

References:

[a] Berényi, O.; Bakonyi, T.; Derakhshifar, I.; Köglberger, H.; Topolska, G.;Ritter, W.; Pechhacker, H.; Nowotny, N. Phylogenetic analysis of Deformed wing virus genotypes from diverse geographic origins indicates recent global distribution of the virus. Appl. Environ. Microbiol. **2007**, *73*, 3605, DOI: 10.1128/AEM.00696-07.

[b] Bradford, E.; Christie, C.R.; Campbell, E.M.; Bowman, A.S. A real-time PCR method for quantification of the total and major variant strains of the deformed wing virus. PLoS ONE **2017**, *12*, DOI 10.1371/journal.pone.0190017.
[c] de Miranda, J.R.; Cordoni, G.; Budge, G. The Acute bee paralysis virus-Kashmir bee virus-Israeli acute paralysis virus complex. *J. Invertebr. Pathol.* **2010**, *103*, S30-S47, DOI 10.1016/j.jip.2009.06.014.
[d] Grabensteiner, E.; Bakonyi, T.; Ritter, W.; Pechhacker, H.; Nowotny, N. Development of a multiplex RT-PCR for the simultaneous detection of three viruses of the honeybee (*Apis mellifera* L.): *Acute bee paralysis virus*, *Black queen cell virus* and *Sacbrood virus*. J. Invert. Pathol. **2007**, *94*, 222-225, DOI: 10.1016/j.jip.2006.11.006.
[e] Tapaszti, Z.; Forgách, P.; Kővágó, C.; Topolska, G.; Nowotny, N.; Rusvai, M.; Bakonyi. T. Genetic analysis and phylogenetic comparison of Black queen cell virus genotypes. Vet. Microbiol. 2009, 139, 227-234, DOI: 10.1016/j.vetmic.2009.06.002.
[f] Grabensteiner, E.; Ritter, W.; Carter, M. J.; Davison, S.; Pechhacker, H.; Kolodziejek, J.; Boecking, O.; Derakhshifar, I.; Moosbeckhofer, R.; Licek, E.; Nowotny, N. Sacbrood virus of the honeybee (Apis mellifera): Rapid identification and phylogenetic analysis using reverse transcription-PCR. Clin. Diagn. Lab. Immunol. **2001**, *8*, 93, DOI: 10.1128/CDLI.8.1.93-104.2001.
[g] Cornman, R.S.; Schatz, M.C.; Johnston, J.S.; Chen, Y.-P.; Pettis, J.; Hunt, G.; Bourgeois, L.; Elsik, C.; Anderson, D.; Grozinger, C.M.; Evans, J.D. Genomic survey of the ectoparasitic mite Varroa destructor, a major pest of the honey bee *Apis mellifera*. BMC Genomics 2010, 11, 602, DOI: 10.1186/1471-2164-11-602.
[h] Runckel, C.; Flenniken, M.L.; Engel, J.C.; Ruby, J.G.; Ganem, D.; Andino, R.; DeRisi, J.L. Temporal analysis of the honey bee microbiome reveals four novel viruses and seasonal prevalence of known viruses, *Nosema*, and *Crithidia*. PLoS ONE **2011**, *6*, e20656, DOI: 10.1371/journal.pone.0020656.
[i] Ravoet, J.; Maharramow, J.; Meeus, I.; De Smet, L.; Wenseleers, T.; Smagghe, G.; de Graaf, D.C. Comprehensive bee pathogen screening in Belgium reveals *Crithidia mellificae* as a new contributory factor to winter mortality. PLoS ONE **2013**, *8*, e72443, DOI: 10.1371/journal.pone.0072443.
[j] Gauthier, L.; Ravallec, M.; Tournaire, M.; Cousserans, F.; Bergoin, M.; Dainat, B.; de Miranda, J.R. Viruses associated with ovarian degeneration in *Apis mellifera* L. queens. *PloS ONE* **2011**, *6*, e16217, DOI 10.1371/journal.pone.0016217.
[k] Palacios, G.; Hui, P.; Quan, P.L.; Kalkstein, A.; Honkavuori, K.S.; Bussetti, A.V.; Conlan, S.; Evans, J.; Chen, Y.P.; vanEngelsdorp, D.; Efrat, H.; Pettis, J.; Cox-foster, D.; Holmes, E.C.; Briese, T.; Lipkin, W.I. Genetic analysis of Israel acute paralysis virus: Distinct clusters are circulating in the United States. J. Virol. **2008**, *82*, 6209, DOI: 10.1128/JVI.00251-08.
[l] Blanchard, P.; Olivier, v.; Iscache, A.-L.; Celle, O.; Schurr, F.; Lallemand, C.; Ribière, M. Improvement of RT-PCR detection of chronic bee paralysis virus (CBPV) required by the description of genomic variability in French CBPV isolates. J. Invert. Pathol. **2008**, *97*, 182-185, DOI: 10.1016/j.jip.2007.07.003.
[m] Blanchard, P.; Schurr, F.; Olivier, V.; Celle, O.; Antùnez, K.; Bakonyi, T.; Berthoud, H.; Haubruge, E.; higes, M.; Kasprzak, S.; Koeglberger, H.; Kryger, P.; Thiéry, R.; Ribière, M. Phylogenetic analysis of the RNA-dependent RNA polymerase (RdRp) and a predicted structural protein (pSP) of the Chronic bee paralysis virus (CBPV) isolated from various geographic regions. Vir. Res. **2009**, *144*, 334-338, DOI: 10.1016/j.virusres.2009.04.025.
