# Supplementary material for: Multiple Virus Infections in Western Honeybee (Apis mellifera L.) Ejaculate Used for Instrumental Insemination
Source: Viruses. 2019 Mar 29;11(4):306. doi: 10.3390/v11040306 (PMC6521257; doi:10.3390/v11040306)
Supplement: Supplementary file 1 [file viruses-11-00306-s001.zip › viruses-467571 supplementary proof/Table S2.pdf]

Table S2: Accession numbers of BQCV and SBV strains used in phylogenetic analyses.

Strain	Virus	Country of origin	GenBank accession No.
Dol826	BQCV	Czech Republic	MG584538
Dol35	BQCV	Czech Republic	MH992095
Dol45	BQCV	Czech Republic	MH992096
Dol47	BQCV	Czech Republic	MH992097
PP	BQCV	Czech Republic	KY243932
R4LY 62	BQCV	Great Britain	GU903466
R4LY 65	BQCV	Great Britain	GU903467
Rothamsted	BQCV	Great Britain	AF125252
Hungary-10	BQCV	Hungary	EF517515
GS1	BQCV	China	KY741959
JL1	BQCV	China	KP119603
KM-1	BQCV	China	MG799370
BQCV_2	BQCV	Russia	KC138853
15A	BQCV	Turkey	FJ588532
POLAND-4	BQCV	Poland	EF517519
QLD	BQCV	Australia	KY465686
SA	BQCV	Australia	KY465685
Sydney	BQCV	Australia	MF623171
South African	BQCV	South Africa	AF183905
AY	BQCV	South Korea	JX149531
Dol51	SBV	Czech Republic	MK550481
Serbia S1	SBV	Serbia	KM001901
155/1030/1253	SBV	Austria	KC513758
1003	SBV	Austria	KC513760
1006	SBV	Austria	KC513759
20100203/24	SBV	France	KC513754
D12-2024/S 1861	SBV	France	KC513753
AFSSA P12	SBV	France	AY230517
Germany 2	SBV	Germany	AF284619
Germany 8	SBV	Germany	AF284625
CSBV-LN/China/2009	SBV	China	HM237361
CSBV-JL/China/2014	SBV	China	KU574661
TN-1	SBV	India	KX663835
II-9	SBV	India	JX270797
BG	SBV	Vietnam	KX668141
AmSBV-Viet4	SBV	Vietnam	KM884993
