# Supplementary material for: Multiple Virus Infections in Western Honeybee (Apis mellifera L.) Ejaculate Used for Instrumental Insemination
Source: Viruses. 2019 Mar 29;11(4):306. doi: 10.3390/v11040306 (PMC6521257; doi:10.3390/v11040306)
Supplement: Supplementary file 1 [file viruses-11-00306-s001.zip › viruses-467571 supplementary proof/Table S3.pdf]

Table S3: List and description of samples and detection of viral sequences in sperm used for insemination

Sample No.	Origin	Condition of colony	The presence of viral sequences					Infection
			DWV	ABPV	BQCV	SBV	*Am*FV	
815/16	Czechia	app. healthy	-	-	-	-	+	single
816/16	Germany	app. healthy	-	-	-	-	-	free
817/16	Germany	app. healthy	+	-	-	-	+	multiple
818/16	Czechia	app. healthy	+	-	-	-	-	single
825/16	Czechia	app. healthy	+	-	-	-	+	multiple
826/16[1]	Czechia	app. healthy	+	-	+	-	+	multiple
827/16	Germany	app. healthy	+	-	-	-	+	multiple
828/16	Germany	app. healthy	+	-	-	-	+	multiple
829/16	Germany	app. healthy	-	-	-	-	+	single
830/16	Czechia	app. healthy	+	-	-	-	+	multiple
831/16	Czechia	app. healthy	+	-	-	-	+	multiple
832/16	Czechia	app. healthy	+	-	+	-	+	multiple
833/16	Austria	app. healthy	-	-	-	-	-	free
834/16	Austria	app. healthy	-	-	-	-	-	free
835/16	Hungary	app. healthy	+	+	-	-	+	multiple
836/16	Hungary	app. healthy	+	+	-	-	+	multiple
837/16	Hungary	app. healthy	+	+	-	-	+	multiple
838/16	Czechia	app. healthy	-	-	-	-	+	single
839/16	Czechia	app. healthy	+	-	-	-	+	multiple
35/17[2]	Czechia	app. healthy	+	-	+	-	+	multiple
37/17	Czechia	app. healthy	-	-	-	-	-	free
38/17	Czechia	app. healthy	+	+	-	-	+	multiple
40/17	Czechia	app. healthy	+	-	-	-	-	single
41/17	Czechia	app. healthy	+	-	-	-	+	multiple
42/17	Czechia	app. healthy	+	-	-	-	+	multiple
43/17	Czechia	app. healthy	+	-	-	-	+	multiple
44/17	Czechia	app. healthy	+	-	-	-	+	multiple
45/17[3]	Czechia	app. healthy	+	-	+	-	+	multiple
46/17	Czechia	app. healthy	+	-	-	-	+	multiple
47/17[4]	Czechia	app. healthy	+	+	+	-	+	multiple
48/17	Czechia	app. healthy	+	-	-	-	-	single
49/17	Czechia	app. healthy	+	+	-	-	+	multiple
50/17	Czechia	app. healthy	+	+	-	-	+	multiple
51/17	Czechia	app. healthy	+	+	-	+	+	multiple
52/17	Czechia	app. healthy	+	-	-	-	-	single
53/17	Czechia	app. healthy	+	-	-	-	-	single
54/17	Czechia	app. healthy	+	-	-	-	-	single
55/17	Czechia	app. healthy	+	-	-	-	-	single
56/17	Czechia	app. healthy	+	-	-	-	-	single
57/17	Czechia	app. healthy	+	-	+	-	+	multiple
58/17	Czechia	app. healthy	+	-	-	-	+	multiple
59/17	Czechia	app. healthy	+	-	-	-	-	single
60/17	Czechia	app. healthy	+	-	-	-	-	single
Total			83.7%	18.6%	13.9%	2.3%	67.4%	

[1]strain Dol826; [2]strain Dol35; [3]strain Dol45; [4]strain Dol47
