# Supplementary material for: Multiple Virus Infections in Western Honeybee (Apis mellifera L.) Ejaculate Used for Instrumental Insemination
Source: Viruses. 2019 Mar 29;11(4):306. doi: 10.3390/v11040306 (PMC6521257; doi:10.3390/v11040306)
Supplement: Supplementary file 1 [file viruses-11-00306-s001.zip › viruses-467571 supplementary proof/Table S4.pdf]

Table S4: The distribution of honey bee viruses in single or mixed infections in drone semen samples.

Detected viral pathogens	Number of positive samples (%)
DWV	10 (25.6)
*Am*FV	3 (7.7)
DWV + *Am*FV	13 (33.3)
DWV + ABPV + *Am*FV	6 (15.4)
DWV + BQCV + *Am*FV	5 (12.8)
DWV + ABPV + BQCV + *Am*FV	1 (2.6)
DWV + ABPV + SBV + *Am*FV	1 (2.6)
Total positive samples	39 (90.7)
